# Prognostic implications of impaired longitudinal left ventricular systolic function assessed by tissue Doppler imaging prior to transcatheter aortic valve implantation for severe aortic stenosis

**DOI:** 10.1007/s10554-021-02519-2

**Published:** 2022-01-10

**Authors:** Guglielmo Gallone, Francesco Bruno, Teresa Trenkwalder, Fabrizio D’Ascenzo, Fabian Islas, Pier Pasquale Leone, Philipp Nicol, Costanza Pellegrini, Enrico Incaminato, Pilar Jimenez-Quevedo, Hector Alfonso Alvarez-Covarrubias, Renato Bragato, Alessandro Andreis, Stefano Salizzoni, Mauro Rinaldi, Adnan Kastrati, Federico Conrotto, Michael Joner, Giulio Stefanini, Luis Nombela-Franco, Erion Xhepa, Javier Escaned, Gaetano M. De Ferrari

**Affiliations:** 1 Division of Cardiology, Department of Medical Sciences, Città della Salute e della Scienza, University of Turin, Corso Bramante 88/90, 10126 Turin, Italy; 2 Deutsches Herzzentrum München, Munich, Germany; 3 Hospital Clínico San Carlos, IDISSC, and Universidad Complutense de Madrid, Madrid, Spain; 4 Humanitas Clinical and Research Center IRCCS, Rozzano-Milan, Italy

**Keywords:** Aortic stenosis, Transcatheter aortic valve replacement, Tissue Doppler imaging, Longitudinal systolic function, Peak systolic mitral annular velocity, Risk prediction

## Abstract

Change in longitudinal left ventricular (LV) systolic function serves as an early marker of the deleterious effect of aortic stenosis (AS) and other cardiac comorbidities on cardiac function. We explored the prognostic value of tissue Doppler imaging (TDI)-derived longitudinal LV systolic function, defined by the peak systolic average of lateral and septal mitral annular velocities (average S’) among symptomatic patients with severe AS undergoing transcatheter aortic valve implantation (TAVI). 297 consecutive patients with severe AS undergoing TAVI at three european centers with available average S’ at preprocedural echocardiography were retrospectively included. The primary endpoint was the Kaplan Meier estimate of all-cause mortality. After a median 18 months (IQR 12–18) follow-up, 36 (12.1%) patients had died. Average S’ was associated with all-cause mortality (per 1 cm/sec decrease: HR 1.29, 95%CI 1.03–1.60, p = 0.025), the cut-off of 6.5 cm/sec being the most accurate. Patients with average S’ < 6.5 cm/sec (55.2%) presented characteristics of more advanced LV remodeling and functional impairment along with higher burden of cardiac comorbidities, and experienced higher all-cause mortality (17.6% vs. 7.5%, p = 0.007), also when adjusted for in-study outcome predictors (adj-HR: 2.69, 95%CI 1.22–5.93, p = 0.014). Results were consistent among patients with preserved ejection fraction, normal-flow AS, high-gradient AS and in those without LV hypertrophy. Longitudinal LV systolic function assessed by average S’ is independently associated with long-term all-cause mortality among TAVI patients. An average S’ below 6.5 cm/sec best defines clinically meaningful reduced longitudinal systolic function and may aid clinical risk stratification in these patients.

**Supplementary Information:**

The online version contains supplementary material available at 10.1007/s10554-021-02519-2.

## Introduction

Left ventricular (LV) responses to aortic valve stenosis (AS) are associated with impaired prognosis. The chronic increase in afterload imposed by AS leads to LV remodeling to counteract the elevated wall stress [1]. When LV hypertrophy is unable to fully compensate for the pressure overload, reduced longitudinal shortening ensues with impairment of myocardial contractility, well before overt global systolic dysfunction becomes apparent [2]. The described response is highly heterogenous, influenced by factors including sex, arterial systemic hypertension, coronary artery disease and amyloidosis, which are highly prevalent in AS and may interact with the clinical benefit of aortic valve replacement [3–6].

By integrating information on LV responses to AS and concomitant cardiac comorbidities, longitudinal systolic function may provide important prognostic value in patients with AS in whom transcatheter aortic valve implantation (TAVI) is being considered as a treatment [7]. While this concept has been proven for speckle tracking-derived systolic global longitudinal strain [8, 9], no study explored the prognostic role of tissue Doppler imaging (TDI)-derived longitudinal systolic function among symptomatic patients with severe AS prior to TAVI.

TDI-derived peak systolic velocity at the mitral annulus (S’) is a widely available index with numerous advantages for the outlined purpose: it is easily obtainable and highly reproducible, sensitive to early longitudinal contractile dysfunction [10], and with demonstrated prognostic predictive value in cardiac conditions including coronary artery disease, mitral regurgitation and heart failure with preserved ejection fraction (EF) [10–12]. Furthermore, the use of S’ may help in circumventing several limitations of speckle tracking-derived strain parameters, including difficulty of application, inter-vendor reference values heterogeneity and inter-observer variability [13], making it a powerful bed-side tool for the clinician.

The present study aims to characterize the clinical and echocardiographic correlates of TDI-derived peak systolic velocity at the mitral annulus and to assess its prognostic value among unselected symptomatic patients with severe AS undergoing TAVI.

## Methods

### Study design

Unselected consecutive patients with severe AS undergoing TAVI from January 2017 to December 2018 at three international Tertiary Centers (Deutsches Herzzentrum München, Munich, Germany; Hospital Clinico San Carlos, Madrid, Spain; Città della Salute e della Scienza Hospital, Turin, Italy) with available TDI-derived longitudinal systolic function measurements at preprocedural echocardiography were retrospectively included in this study.

TAVI was performed according to local expertise and standard techniques. All patients provided written informed consent before the procedure. The registry was approved by the local ethics committee and was conducted in accordance with the Declaration of Helsinki.

### Echocardiographic assessment and data collection

Baseline clinical, echocardiographic and laboratory variables along with clinical follow-up data were prospectively collected at each institution and retrospectively analyzed. Baseline echocardiography was performed in all patients within 3 months before the TAVI procedure. When available, echocardiographic follow-up data were also collected. The echocardiographic evaluation was independently performed by experienced cardiologists who were blinded to patient outcomes.

**Fig. 1:**
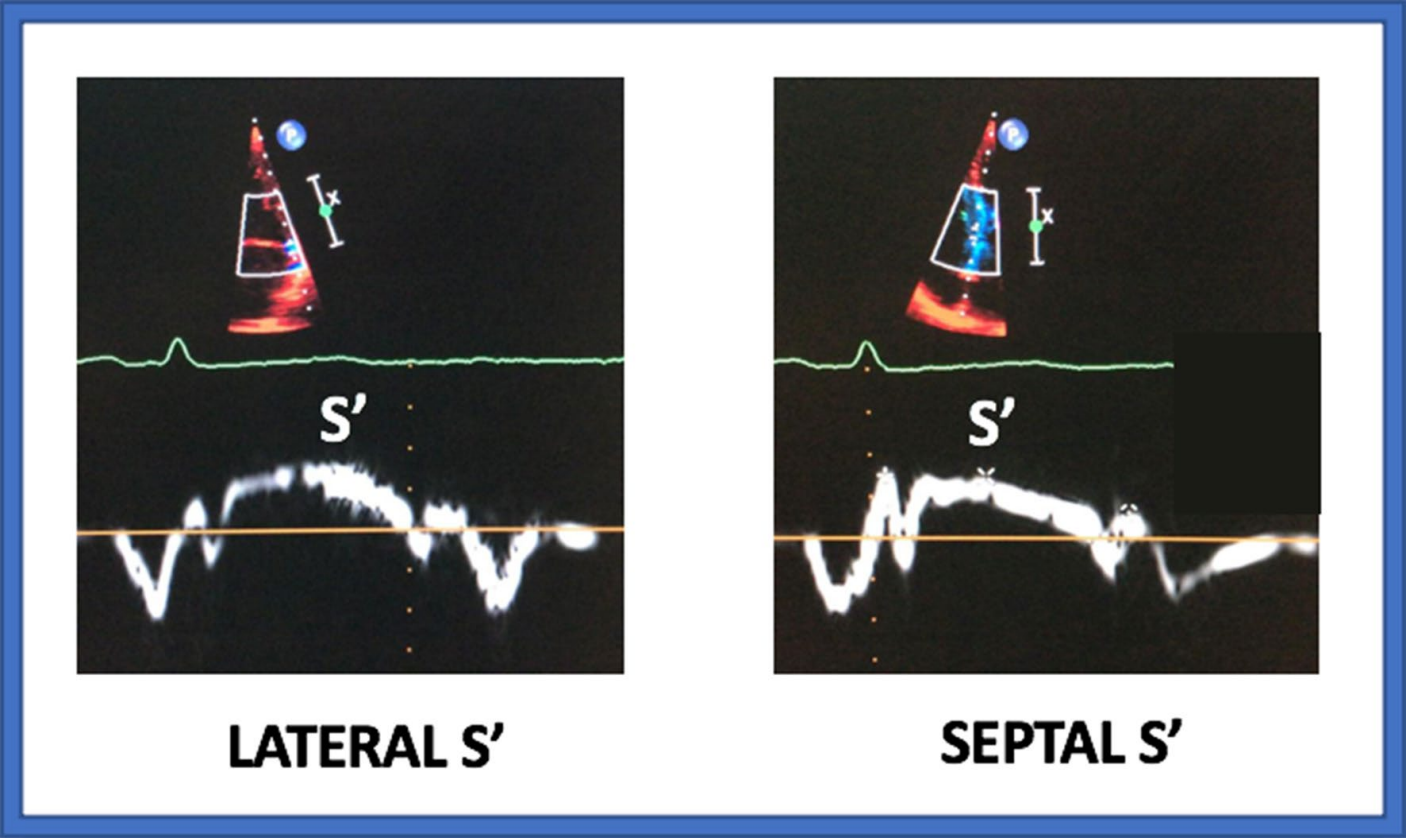
Measurement of peak systolic (S’) velocities by 2-dimensional tissue Doppler imaging. S’ represents the peak myocardial longitudinal systolic shortening velocity during ejection assessed by TDI. S′ velocities are measured at the level of the lateral (left panel) and medial (right panel) mitral annulus by pulsed-wave TDI from the apical four-chamber view. Average S’ is calculated as the mean of the lateral and medial S’ velocities. TDI is performed adjusting gain and frame rate to get an appropriate tissue characterization

Echocardiographic exams were performed according to the guidelines of the American Society of Echocardiography [14]. LV volumes and LV mass were determined utilizing standard techniques. LV EF was calculated measuring volumes with a biplane measurement from the apical views using the modified Simpson’s method. Trans-mitral early (E) and late (A) velocities and E wave deceleration time were measured by spectral pulsed-wave Doppler ultrasound at the mitral leaflet tips. TDI was performed adjusting gain and frame rate to get an appropriate tissue characterization. Peak systolic (S′) and early (E′) velocities of the lateral and medial mitral annulus were measured by pulsed-wave TDI from the apical four-chamber view and the average was calculated (Fig. 1). Diastolic dysfunction was evaluated and graded according to the guidelines of the American Society of Echocardiography [14]. The aortic valve area was calculated by the continuity equation, and the maximum pressure gradient across the restrictive orifice was estimated by the modified Bernoulli equation. Mean transaortic pressure gradient was calculated averaging the instantaneous gradients over the ejection period on the continuous-wave Doppler recordings. LV stroke volume was calculated multiplying the systolic velocity–time integral at the LV outflow tract per its area and was indexed to body surface area (SVi). The severity of valvular regurgitation was determined on a qualitative scale (mild, moderate, and severe), according to the current guidelines for the management of patients with valvular heart disease [15].

The Society for Thoracic Surgery Predictive Risk of Mortality (STS PROM) score [16] and the EuroSCORE II [17] were prospectively calculated.

### Study endpoints

The primary endpoint was all-cause mortality at follow-up. Secondary endpoints were a composite of all-cause mortality or hospitalization for heart failure (HF) at last follow-up and Valve Academic Research Consortium (VARC)-2 defined adverse outcomes assessed at 30 days [18].

### Statistical analysis

Categorical variables are expressed as number and percentages, continuous variables are expressed as mean ± standard deviation or median and interquartile range (IQR) as appropriate. Unpaired t test or nonparametric Mann–Whitney U test were used for comparisons of continuous variables and chi-square test was used for categorical variables. Peak systolic average of lateral and septal mitral annular velocities (average S’) was analyzed both continuously and at the best cut-off value to predict the primary endpoint determined by Receiver Operating Characteristic (ROC) curve analysis applying the Youden’s J statistic.

Kaplan–Meier and Cox proportional hazard models were performed to evaluate cumulative event rates of the primary endpoint at long-term follow-up and results are presented as hazard ratio (HR) and 95% confidence intervals (CIs). To produce meaningful outcome estimates, maximum follow-up length was truncated at 18 months (corresponding to the 50th percentile of available follow-up length in the study population).

A multivariate Cox proportional hazards analysis was performed to assess the independent association of average S’ with all-cause mortality, and all-cause mortality or HF hospitalization. All the variables with a univariate p < 0.10 were entered into the models.

Subgroups analyses were carried according to relevant variables (EF: < 50% vs ≥ 50%; stroke volume index [SVi]: < 35 ml/m^2^ vs ≥ 35 ml/m^2^, mean transvalvular gradient: < 40 mmHg vs ≥ 40 mmHg, LV hypertrophy: LV mass index ≥ 115 gr/m2 for male, ≥ 95 gr/m2 for female vs < 115 gr/m^2^ for male, < 95 gr/m^2^ for female).

An exploratory analysis was carried in a subgroup of patients with available post-TAVI discharge echocardiography to assess the prognostic implications of changes in average S’.

A p < 0.05 was considered statistically significant. Statistical analyses were conducted using SPSS (version 24.0, SPSS Inc., Chicago, Illinois, US).

## Results

### Study population and outcomes

Overall, 297 unselected patients with severe AS undergoing TAVI and with available TDI-derived longitudinal systolic function measurements constituted the study population. Baseline clinical and echocardiographic characteristics are described in Tables 1 and 2.

**Table 1:** Distribution of baseline clinical characteristics in the overall study population and stratified by average S’ status

	Overall population (n = 297)	Average S' < 6.5 cm/s (n = 164)	Average S' ≥ 6.5 cm/s (n = 133)	p-value
Age (y)	81 ± 6	82 ± 5	80 ± 6	0.898
Female Sex (%)	150 (50.5)	84 (51.2)	66 (49.6)	0.816
BMI	27.0 ± 4.8	26.6 ± 4.9	27.6 ± 4.6	0.907
Cardiovascular Risk Factors				
Smoker (%)	31 (10.4)	15 (9.1)	16 (12)	0.243
Hypertension (%)	257 (86.5)	144 (87.8)	113 (85)	0.498
Diabetes (%)	81 (27.3)	41 (25)	40 (30.1)	0.360
Dyslipidemia (%)	185 (65.1)	105 (65.2)	80 (65)	1.000
Medical History				
Known CAD (%)	143 (51.1)	74 (48.1)	69 (54.8)	0.281
Prior MI (%)	42 (14.1)	32 (19.5)	10 (7.5)	0.004
Prior PCI (%)	75 (25.3)	42 (25.6)	33 (24.8)	0.894
Prior CABG (%)	18 (6.5)	10 (6.5)	8 (6.5)	1.000
Prior stroke (%)	43 (14.5)	25 (15.2)	18 (13.5)	0.742
Previous aortic valve replacement (%)	7 (2.4)	3 (1.8)	4 (3)	0.704
Known PAD (%)	28 (16.3)	20 (21.7)	8 (10)	0.041
Known Atrial Fibrillation (%)	108 (36.4)	74 (45.1)	34 (25.6)	0.001
Clinical Characteristics				
Sistolic BP (mmHg)	139 ± 20	134 ± 19	146 ± 19	0.758
Diastolic BP (mmHg)	76 ± 11	75 ± 11	78 ± 10	0.365
Heart Rate (bpm)	70 ± 13	72 ± 13	68 ± 12	0.268
NYHA Class (%)				
1	38 (12.8)	17 (10.4)	21 (15.8)	
2	98 (33.1)	55 (33.7)	43 (32.3)	
3	146 (49.3)	83 (50.9)	63 (47.4)	
4	14 (4.7)	8 (4.9)	6 (4.5)	0.284
COPD (%)	33 (11.8)	19 (12.3)	14 (11.1)	0.853
Any conduction disturbance (%)	98 (37.1)	54 (37.8)	44 (36.4)	0.898
PM/ICD at baseline (%)	31 (11)	22 (14.1)	9 (7.1)	0.084
Hb (gr/dL)	12.5 ± 1.8	12.4 ± 1.8	12.5 ± 1.8	0.824
eGFR (Cockroft-Gault)	55.2 ± 16.4	53.1 ± 15.1	57.9 ± 17.6	0.056
Dialysis (%)	5 (1.8)	3 (1.9)	2 (1.6)	1.000
HsTn (URL-fold)	1.35 (0.5—2.5)	1.4 (0.7—2.4)	1.3 (0.3—2.8)	0.335
NT-proBNP (ng/dL)	1820 (615–4418)	2370 (776–5121)	1380 (532–3630)	0.157
Risk Assessment				
EuroSCORE II	4.3 (2.6—7.2)	4.9 (2.8—8.3)	3.7 (2.3—5.9)	0.051
STS-PROM	4.4 (2.8—6.7)	4.6 (3.2—7.0)	3.9 (2.2—6.4)	0.365
Implanted prosthesis				
Balloon-expandable (%)	144 (48.5)	80 (48.8)	64 (48.1)	
Self-expanding (%)	153 (51.5)	84 (51.2)	69 (51.9)	1.000

Values are expressed as n/N of patients (%) or mean ± standard deviation or median and interquartile range

*AV* aortic valve, *BMI* body mass index, *CABG* coronary artery bypass grafting, *CAD* coronary artery disease, *COPD* chronic obstructive pulmonary disease, *eGFR* estimated glomerular filtration rate, *ICD* implantable cardioverter defibrillator, *Hb* haemoglobin, *HsTn* high-sensitivity troponin, *STS PROM* Society for Thoracic Surgery Predictive Risk of Mortality, *MI* myocardial infarction; *NYHA* New York Heart Association, *PCI* percutaneous coronary intervention, *PAD* peripheral artery disease, *PM* pacemaker, *TAVI* transcatheter aortic valve implantation, *URL* Upper reference limit

**Table 2:** Distribution of baseline echocardiographic characteristics in the overall study population and stratified by average S’ status

	Overall population (n = 297)	Average S' < 6.5 cm/s (n = 164)	Average S' ≥ 6.5 cm/s (n = 133)	p-value
Aortic Valve				
Max AV gradient (mmHg)	79.1 ± 21.4	70.1 ± 23.9	74.7 ± 17.3	0.003
Mean AV gradient (mmHg)	43.5 ± 13.7	42.1 ± 15.1	45.3 ± 11.6	0.015
AVA (cm2)	0.7 ± 0.2	0.67 ± 0.2	0.75 ± 0.2	0.633
SVi (ml/m2)	39.3 ± 13.2	36.9 ± 12.1	42.2 ± 14.0	0.600
Left Ventricle				
LVEF (%)	55.9 ± 13.0	52.5 ± 14.5	60.1 ± 9.4	< 0.001
iLVESV (ml/m2)	28.8 ± 20.9	33.2 ± 23.3	21.6 ± 13.5	< 0.001
iLVEDV (ml/m2)	59.8 ± 24.9	63.4 ± 26.1	53.2 ± 21.2	0.154
iTSD (mm/m2)	19.1 ± 5.6	20.2 ± 5.6	17.5 ± 5.2	0.535
iTDD (mm/m2)	26.1 ± 5.1	27.3 ± 5.5	24.7 ± 4.2	0.016
IVS thickness (mm)	13.6 ± 2.3	13.9 ± 2.6	13.3 ± 2.1	0.134
PW thickness (mm)	12.2 ± 2.0	12.3 ± 2.1	12.0 ± 2.0	0.482
LV mass index (g/m2)	128.4 ± 39.9	138.1 ± 41.3	116.7 ± 34.8	0.214
Valves				
AR ≥ moderate (%)	56 (19.2)	33 (20.6)	23 (17.6)	0.552
MR ≥ moderate (%)	73 (24.7)	54 (33.3)	19 (14.3)	< 0.001
TR ≥ moderate (%)	45 (15.4)	35 (21.6)	10 (7.6)	0.001
Diastolic Function				
Diastolic dysfunction	289 (97.6)	163 (99.4)	126 (95.5)	0.032
Diastolic dysfunction ≥ 2 grade	167 (57.8)	110 (63.0)	57 (45.3)	< 0.001
E wave (cm/s)	97.1 ± 34.0	101.4 ± 31.6	91.8 ± 36.0	0.565
E Deceleration Time (s)	0.2 ± 0.1	0.2 ± 0.1	0.2 ± 0.1	0.784
A wave (cm/s)	100.4 ± 35.3	92.5 ± 36.1	109.1 ± 32.6	0.293
E/A ratio	1.1 ± 0.9	1.3 ± 1.1	0.9 ± 0.4	< 0.001
Lateral e’ (cm/s)	6.7 ± 2.2	6.6 ± 2.3	6.9 ± 2.2	0.561
Septal e’ (cm/s)	236 (89.1)	136 (91.9)	100 (85.5)	0.114
Average e’ (cm/s)	6.1 ± 1.9	5.8 ± 1.8	6.4 ± 1.9	0.988
E/e’ ratio	17.2 ± 7.6	18.8 ± 7.8	15.1 ± 6.7	0.141
TDI-derived longitudinal systolic function				
Septal mitral annular S’ (cm/s)	5.7 ± 1.7	4.7 ± 0.9	7.0 ± 1.5	0.001
Lateral mitral annular S’ (cm/s)	6.9 ± 1.7	5.8 ± 1.2	8.2 ± 1.3	0.080
Average mitral annular S’ (cm/s)	6.2 ± 1.6	5.1 ± 0.9	7.6 ± 1.1	0.259
Others				
LAVi (ml/m2)	49.9 ± 20.1	53.5 ± 22.2	45.3 ± 16.2	0.010
sPAP (mmHg)	38.3 ± 14.5	42.0 ± 15.7	33.8 ± 11.3	0.001
TAPSE (mm)	20.5 ± 4.9	19.6 ± 4.4	21.6 ± 5.4	0.239
Pericardial effusion (%)	31 (11)	17 (10.8)	14 (11.3)	1.000
CVP (%)				
3	166 (83)	87 (77.7)	79 (89.8)	
8	13 (6.5)	10 (8.9)	3 (3.4)	
15	21 (10.5)	15 (13.4)	6 (6.8)	0.125

Values are expressed as n/N of patients (%) or mean ± standard deviation

*AR* aortic regurgitation, *AV* aortic valve, *AVA* aortic valve area, *CVP* central venous pressure, *iLEDV* indexed left ventricle end-diastolic volume, *iLVESV* indexed left ventricle end-systolic volume, *iTDD* indexed tee-diastolic volume, *iTSD* indexed tele-systolic diameter, *IVS* interventricular septum, *LAVi* left atrial volume indexed, *LVEF* left ventricle ejection fraction, *MR* mitral regurgitation, *sPAP* systolic pulmonary artery pressure, *PW* posterior wall, *TAPSE* tricuspid annular plane systolic excursion, *TDI* tissue doppler imaging, *TR* tricuspid regurgitation, *SVi* stroke volume index

The mean age was 81 ± 6 years and 150 (50.5%) patients were female. Mean STS PROM was 5.5 ± 4.4% and mean Euroscore II was 6.1 ± 5.8%.

At echocardiographic assessment, 25% of the patients had reduced EF (EF < 50%), 74.3% had LV hypertrophy (LV mass index ≥ 115 gr/m^2^ for male, ≥ 95 gr/m^2^ for female), 39.5% had low-flow AS (SVi < 35 ml/mq) and 36.7% had low-gradient AS (mean AS gradient < 40 mmHg).

After a median follow-up of 18 months (IQR 12–18 months), 36 (12.2%) patients died, and 44 (14.8%) died or were hospitalized for HF. Procedural outcomes are reported in Supplementary Table 1 and short-term VARC-2 outcomes are reported in Table 3.

**Table 3:** Short- and Long-Term Clinical Outcomes according to average S’ status

	Average S' < 6.5 cm/s (n = 164)	Average S' ≥ 6.5 cm/s (n = 133)	P-value
	30-day outcomes		
All-cause Death (%)	4 (2.5)	1 (0.8)	0.383
Bleeding (%)			
Life-threatening	3 (1.9)	8 (6)	0.201
Major	10 (6.2)	7 (5.3)	0.424
AKI stage 2–3 (%)	2 (1.2)	3 (2.3)	0.661
Vascular Complications (%)			
Major	15 (9.3)	14 (10.6)	0.910
Minor	19 (11.8)	13 (9.8)	0.736
Myocardial Infarction (%)	0 (0)	1 (0.8)	0.451
Stroke/TIA (%)	3 (1.8)	2 (1.5)	1.000
New Pacemaker (%)	19 (11.8)	20 (15.2)	0.490
Coronary Obstruction (%)	0 (0)	1 (0.8)	0.451
	1-year outcomes		
All-cause Death (%)	18 (11.5)	6 (4.9)	0.055
HF Hospitalitazion (%)	14 (9.1)	17 (13.8)	0.252
Stroke/TIA (%)	4 (2.5)	2 (2.4)	0.639
Disabling stroke	1 (0.6)	1 (0.8)	0.859
Endocarditis (%)	1 (0.6)	4 (3.3)	0.174
THV Thrombosis (%)	3 (1.9)	4 (3.3)	0.703
NYHA class (%)			
1	78 (56.1)	61 (52.6)	
2	56 (40.3)	46 (39.7)	
3	5 (3.6)	8 (6.9)	0.499
	Long-term outcomes		
All-cause Death (%)	28 (17.1%)	8 (6.1%)	0.003
A-cause Death/HF hosp. (%)	31 (19.6)	13 (10.2)	0.020

Values are expressed as n/N of patients (%)

*AKI* acute kidney injury, *THV* transcatheter heart valve. Other abbreviations as in Table 1

### TDI-derived longitudinal left ventricular systolic function

Mean peak systolic average of lateral and septal mitral annular velocities (average S’) was 6.2 ± 1.6 cm/sec. Average S’ was associated with increased all-cause mortality at last follow-up (per 1 cm/sec decrease: HR 1.29, 95% CI 1.03–1.60, p = 0.025). The best average S’ for all-cause mortality was 6.5 cm/sec, obtained from ROC analysis (area under the ROC 0.632, 95%CI 0.538–0.726, p = 0.033).

Patients with reduced average S’ (< 6.5 cm/sec, 55.2% of the study population) had similar clinical characteristics to patients with S’ ≥ 6.5 cm/sec, except for more frequent prior myocardial infarction (19.5% vs 7.5%, p = 0.004) and atrial fibrillation (45.1% vs 25.6%, p = 0.001) (Table 1). At preprocedural echocardiography, patients with reduced average S’ presented with characteristics of more advanced LV remodeling and functional impairment (Table 2). In particular, reduced EF (32.9% vs. 9.2%, p < 0.001), LV hypertrophy (83.5% vs. 60.3%, p < 0.001), low-flow AS (48.0% vs. 31.4%, p = 0.004) and low-gradient AS (42.7% vs. 30.5%, p = 0.021) were more frequent among patients with reduced average S’.

**Fig. 2:**
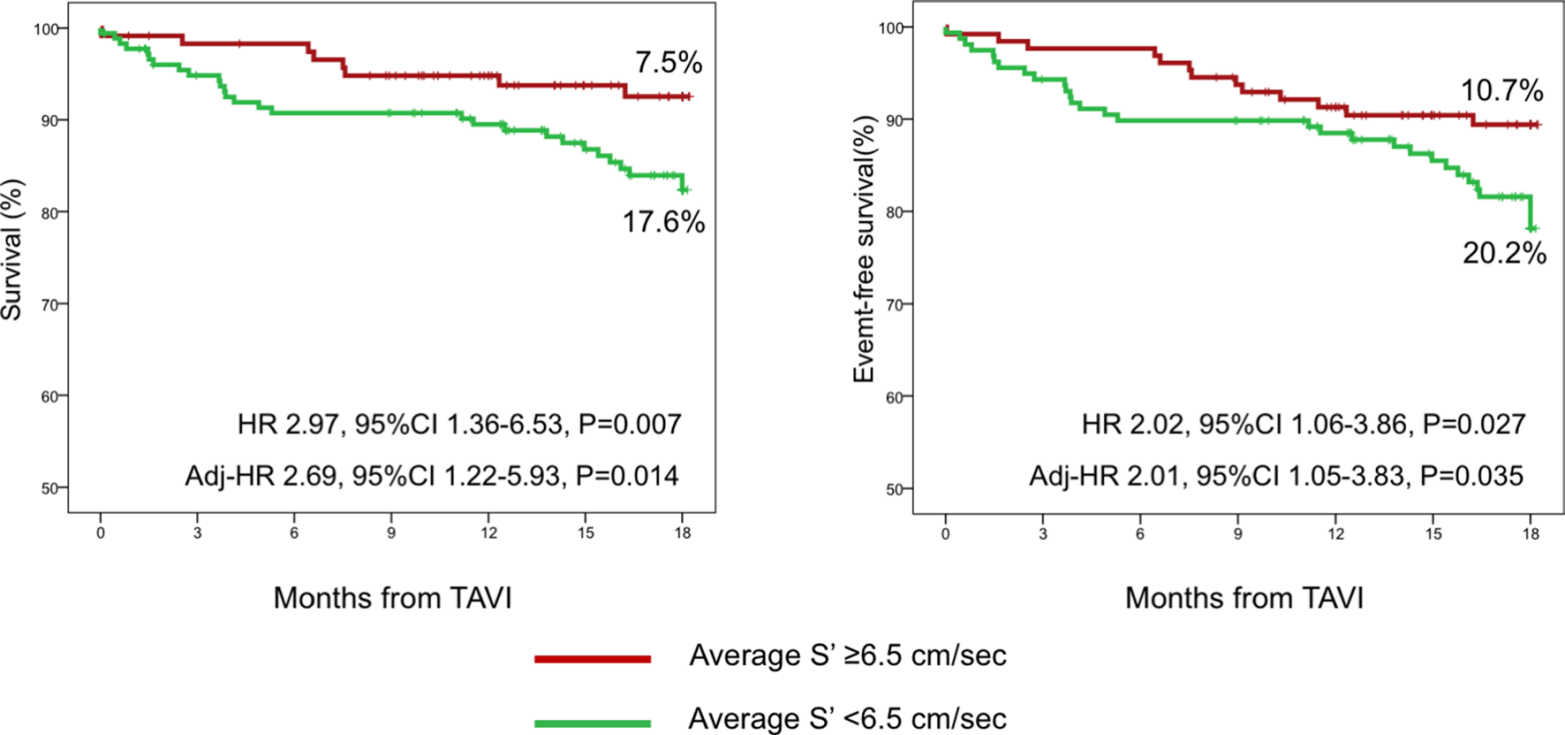
Kaplan Meier curves for all-cause mortality and all-cause mortality or heart failure hospitalization at 540 days follow-up stratified by average S’. Abbreviations as in Tables 1 and 3. Hazard ratios are derived from Cox proportional hazard models

Patients with reduced average S’ had higher Kaplan Meier estimates of all-cause mortality (17.6% vs. 7.5%; HR 2.97, 95%CI 1.36–6.33, p = 0.007) and all-cause mortality or HF hospitalization (20.2% vs. 10.7%; HR 2.02, 95%CI 1.06–3.86, p = 0.027) (Fig. 2). Short-term outcomes were similar regardless of average S’ status (Table 3).

### Predictors for adverse clinical outcomes

Multivariable models of predictors for all-cause mortality and of all-cause mortality or HF hospitalization at last follow-up are presented in Table 4. After multivariable adjustment, reduced average S’ (adj-HR: 2.69, 95%CI 1.22–5.93, p = 0.014) and Euroscore II (per 1% increase: 1.05, 95%CI 1.01–1.09, p = 0.009) were independent predictors of all-cause mortality, while reduced average S’ (Adj-HR 2.01, 95%CI 1.05–3.83, p = 0.035) and mean AS gradient (per 1 mmHg decrease: 1.03, 95%CI 1.01–1.06, p = 0.018) were independent predictors of all-cause mortality or HF hospitalization. Results remain consistent also when further adjusting for TAVI Centers (all-cause mortality: Adj-HR 3.09, 95%CI 1.16–8.20, p = 0.024; all-cause mortality or HF hospitalization: Adj-HR 2.96, 95%CI 1.19–7.41, p = 0.038).

**Table 4:** Predictive factors for long-term all-cause mortality and all-cause mortality or heart failure hospitalization

	All-cause mortality*			
	Univariate		Multivariate	
	HR (95% CI)	p	HR (95% CI)	p
Age (yrs)	1.07 (1.00 ± 1.15)	0.046		
Hb (gr/dL)	0.81 (0.66 ± 0.99)	0.041		
Known atrial fibrillation	2.08 (1.08 ± 4.01)	0.028		
eGFR (Cockroft-Gault)	0.98 (0.96 ± 1.00)	0.056		
Euroscore II (%)	1.06 (1.02 ± 1.10)	0.002	1.05 (1.01–1.09)	0.009
Mean AV gradient (mmHg)	0.98 (0.95 ± 1.00)	0.091		
Septal e' (cm/sec)	0.79 (0.62 ± 1.02)	0.066		
Average S' < 6.5 cm/s	2.38 (1.08 ± 5.23)	0.030	2.69 (1.22–5.93)	0.014
TR ≥ moderate	1.68 (1.15 ± 2.45)	0.007		

	ALL-cause mortality/ HF hospitalization*			
	Univariate		Multivariate	
	HR (95% CI)	p	HR (95% CI)	p
Age (yrs)	1.07 (1.00 ± 1.15)	0.046		
Hb (gr/dL)	0.81 (0.66 ± 0.99)	0.041		
Known atrial fibrillation	2.08 (1.08 ± 4.01)	0.028		
eGFR (Cockroft-Gault)	0.98 (0.96 ± 1.00)	0.056		
Euroscore II	1.06 (1.02 ± 1.10)	0.002		
Mean AV gradient (mmHg)	0.98 (0.95 ± 1.00)	0.091	0.97 (0.94–0.99)	0.018
Septal e' (cm/sec)	0.79 (0.62 ± 1.02)	0.066		
Average S' < 6.5 cm/s	2.38 (1.08 ± 5.23)	0.030	2.01 (1.05–3.83)	0.035
TR ≥ moderate	1.68 (1.15 ± 2.45)	0.007		

*Median follow-up: 540 (363–540) days

*CI* Confidence Interval, *HR* Hazard Ratio. Other abbreviations as in Tables 1 and 2

### Association of reduced average S’ with all-cause mortality in relevant subgroups

**Fig. 3:**
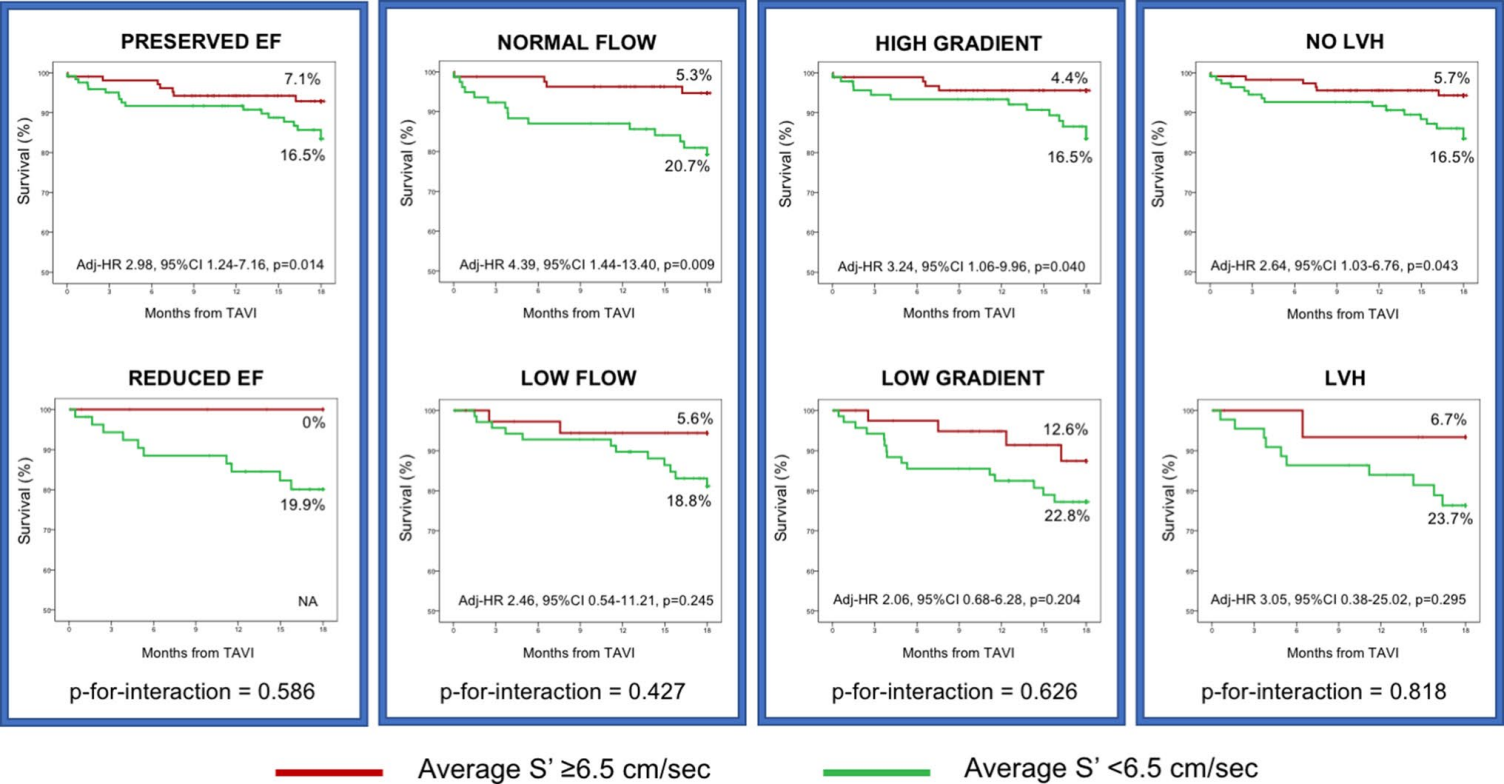
Association of average S’ with all-cause mortality across subgroups. Black squares represent hazard ratios (HR); horizontal black lines illustrate CI. Reduced EF: EF < 50%, low flow: stroke volume index < 35 ml/m^2^, low gradient: mean transvalvular gradient < 40 mmHg, LVH: LV mass index ≥ 115 gr/m2 for male, ≥ 95 gr/m2 for female. *Interaction P testing for an effect modification of the subgroups on the difference in all-cause death between average S’ < 6.5 cm/sec vs. ≥ 6.5 cm/sec. Abbreviations as in Tables 1 and 2

Kaplan Meier estimates for the primary endpoint stratified by average S’ among subgroups of EF, SVi, AS gradient and LV hypertrophy are presented in Fig. 3. A reduced average S’ remained independently associated with all-cause mortality among patients with less severe structural remodeling and functional impairment (preserved EF subgroup: adj-HR 2.98, 95%CI 1.24–7.16, p = 0.014; normal flow subgroup: adj-HR 4.39, 95%CI 1.44–13.40, p = 0.009; high gradient AS subgroup: adj-HR 3.24, 95%CI 1.06–9.96, p = 0.040; no-LV hypertrophy subgroup: adj-HR 2.64, 95%CI 1.03–6.76, p = 0.043).

### Prognostic relevance of average S’ improvement following TAVI

156 (52.5%) patients had available post-TAVI echocardiography with average S’ measurements (median 64 [IQR 42–83] days post-TAVI).

Of 80 patients with reduced average S’ pre-TAVI, 40 (50%) had average S’ “normalization” (≥ 6.5 cm/sec) following TAVI. Patients experiencing average S’ normalization had non significantly different all-cause mortality estimates as compared to patients with normal average S’ pre-TAVI (13.8% vs. 7.4%, log-rank = 0.263), while patients with persistently reduced average S’ had significantly higher all-cause mortality estimates (22.6% vs. 7.4%, p = 0.043).

## Discussion

The main finding of our study is that, in patients with severe AS undergoing TAVI, impairment of longitudinal LV systolic function, estimated by average S’ measurements, predicts medium-term all-cause mortality. The association between average S´ and mortality was independent from the patient’s clinical profile and from the echocardiographic parameters of cardiac structure and function, and it was consistently observed among patient subgroups with less severe structural remodeling or functional impairment (including those with preserved EF, normal SVi, high gradient AS or no LV hypertrophy). For clinical purposes, an average S’ below 6.5 cm/sec best defines clinically meaningful reduced longitudinal LV systolic function in symptomatic severe AS.

This is the first study assessing the prognostic value of average S’ among symptomatic patients with severe AS undergoing TAVI. A previous study by Stewart et al. tested the role of average S’ among 183 asymptomatic patients with moderate to severe AS, failing to demonstrate independent predictive value for clinical deterioration after accounting for the severity of AS [4]. In the aforementioned study, average S’ was higher than in our population (median [IQR]: 6.7 [6.0–7.9] cm/sec vs. 6.1 [5.1–7.3] cm/sec) coherently with the different phases of AS natural history. Indeed, the study by Stewart et al. comprised patients in an earlier phase of AS progression, which is likely to have imposed less severe loading conditions and for less time, resulting in a milder morpho-functional LV adverse remodelling. Major differences between both studies may explain the discrepancy in the results. In addition to a much larger study population (n = 297), our study included only symptomatic patients with severe AS eligible for TAVI, while in the study by Stewart only 62% eventually underwent surgical aortic valve replacement. Moreover, mechanisms of reduced longitudinal contractility may vary over the natural history of AS. Indeed, the observation that the association of average S′ with clinical deterioration was fully accounted by AS severity among asymptomatic moderate to severe AS patients suggests that, when afterload-mismatch is the mechanism of decreased longitudinal systolic function, no prognostic implication ensues [4]. Conversely, in more advanced AS disease, maladaptive mechanisms may underlie the longitudinal systolic function impairment entailing worse outcome despite valve replacement [19, 20].

Average S’ was the sole prognostic echocardiographic predictor along with the patient’s clinical risk profile as assessed by the Euroscore II tool. This finding mirrors those of a prior study in which longitudinal systolic function as assessed by global longitudinal strain recapitulated the mortality impact of all other myocardial and valve structural and functional echocardiographic parameters [21]. From a clinical perspective, the implications of our results are two-folded. On one side, average S’ emerges as a powerful indicator that may aid mortality prediction following TAVI, an important goal to improve physician–patient communication and to orientate clinical decision making. On the other side, as average S’ also predicts the composite of mortality or HF hospitalizations, it may be a useful tool to identify those patients more prone to HF progression, that may benefit from closer clinical follow-up to assess fluid status and adequacy of diuretic therapy and, where appropriate, initiation and up-titration of disease-modifying medical treatments.

In our study, reduced average S’ was more frequently observed among patients with previous MI, moderate to severe mitral or tricuspid regurgitation and atrial fibrillation. This highlights how average S’ may reflect the summative effect of different pathophysiological mechanisms related to frequently existing conditions in patients with AS that also affect myocardial function. Of note, the information obtained predicts patient prognosis despite valve replacement. A reduced average S’ (< 6 cm/sec) has been proposed as a highly accurate marker (Sensitivity 100%, Specificity 57%) to screen TAVI candidates for transthyretin cardiac amyloidosis, outperforming speckle-strain imaging parameters [3]. This condition may be prevalent in up to 16% patients with severe AS undergoing TAVI [3] and may be associated with worse outcomes following valve replacement [6], an observation that may be indirectly reflected by the findings of the present study.

Our results were consistent among patients with preserved EF, normal SVi or high gradient AS. These AS subgroups are overall associated with better outcomes following TAVI [5, 22–24] and show no apparent pump dysfunction in most patients, especially in case of compensatory LV hypertrophy [25]. Our findings reinforce the concept of impaired longitudinal myocardial shortening as an early marker of systolic dysfunction and establish its prognostic value among symptomatic severe AS patients undergoing TAVI also when isolated reduced average S’ is found.

In exploratory analyses, we evaluated the clinical significance of the change in average S’ following TAVI. Among patients with pre-TAVI reduced average S’, longitudinal systolic function recovered in one out of two patients (post-TAVI average S’ ≥ 6.5 cm/sec). Long-term mortality in patients with recovered average S’ was similar to those of patients with pre-TAVI preserved average S’, while prognosis remained poorer for patients with persistently reduced S’. This finding suggests once again that several mechanisms may underlie reduced contractile function in severe AS, entailing differential prognostic implications [1, 19, 20, 26, 27]. More importantly, it adds up to current literature that demonstrated LV reverse remodeling following TAVI to be a positive marker of favorable long-term outcome [27–29] and it points at post-TAVI average S’ as a reliable echocardiographic feature able to define the patient’s trajectory also within the preserved EF population and at an early assessment, thus providing an advantage over post-TAVI LV mass and EF which recover throughout a longer time course [27]. Since this analysis was carried on a limited proportion of the population with available post-TAVI S’ assessment, it has to be considered hypothesis generating requiring further validation in dedicated studies.

In the present study we assessed longitudinal systolic function by average S’, a TDI-derived parameter of LV long-axis motion measured at the mitral annular level. This approach lacks the ability to reflect segmental functional abnormalities, is affected by signal noise and requires accurate parallel alignment of the Doppler beam with myocardial motion direction. While these drawbacks are at least partly overcome by speckle-tracking echocardiography, whose ability to risk stratify patients in symptomatic severe AS patients has been previously demonstrated [21], the latter is less practical, presents a longer learning curve and requires proprietary software with inter-vendor variability in reference values [13]. To this regard, TDI remains a widely available tool, with great ease of use and high reproducibility which may provide powerful clinical information to orientate risk stratification and patient management across a variety of clinical conditions. Of note, the segmental nature of speckle tracking-derived strain parameters (of clinical relevance across several myocardial diseases) seems to provide limited advantage in severe AS where impaired longitudinal function is primarily reflected at the basal myocardial level. Indeed, basal longitudinal fibers are more exposed to the increased interventricular pressure during isovolumic contraction and are firstly affected by impaired longitudinal shortening as compared to mid-apical segments. This concept is proved on clinical ground, with previous demonstrations of basal longitudinal strain as a more powerful predictor of symptoms and outcomes as compared to global longitudinal strain in AS [30, 31].

## Limitations

The findings of this study should be interpreted in the light of several limitations. First, this was a retrospective registry of clinical practice data. Despite the inherent limitations of study design and missing data, our findings have the advantage of generalizability to the real-world clinical setting. Second, only patients with pre-TAVI TDI assessment were included. Although this may in theory represent a source of selection bias, it is unlikely to be clinically significant. Indeed, the availability of TDI measurements seems to depend on the routine operator practice, rather than dictated by clinical reasons. This is also suggested by the clinical and echocardiographic characteristics of the included TAVI population well mirroring current clinical practice. Third, the study sample size was relatively small. However, this represents the largest available study of average S’ in symptomatic severe AS, with consistent results across study subgroups and a grounded physiopathological rationale. Fourth, we did not assess the independent prognostic impact of average S’ against speckle-tracking derived longitudinal strain. As discussed above, this was not the scope of the present analysis and the findings of the study should be interpreted and applied within the boundaries of the study design.

## Conclusions

TDI-derived peak systolic average of lateral and septal mitral annular velocities is associated with long-term all-cause mortality among unselected patients with symptomatic severe AS undergoing TAVI. In this population, an average S’ below 6.5 cm/sec best defines clinically meaningful reduced longitudinal LV systolic function and may aid clinical risk stratification.

### Supplementary Information

Below is the link to the electronic supplementary material.

Supplementary file1 (docx 19 KB)

## Data Availability

Data will be made available to interested parties by the corresponding author upon reasonable request.

## Abbreviations

AS: Aortic stenosis
CI: Confidence interval
EF: Ejection Fraction
HF: Heart failure
HR: Hazard ratio
IQR: Interquartile Range
LV: Left ventricle
STS PROM: Society for Thoracic Surgery Predictive Risk of Mortality
TAVI: Transcatheter aortic valve implantation
TDI: Tissue Doppler Imaging
VARC: Valve Academic Research Consortium
