# Supplementary material for: Prognostic implications of impaired longitudinal left ventricular systolic function assessed by tissue Doppler imaging prior to transcatheter aortic valve implantation for severe aortic stenosis
Source: Int J Cardiovasc Imaging. 2022 Jan 10;38(6):1317–28. doi: 10.1007/s10554-021-02519-2 (PMC11142981; doi:10.1007/s10554-021-02519-2)
Supplement: Supplementary file 1 — Supplementary file1 (docx 19 KB) [file 10554_2021_2519_MOESM1_ESM.docx]

**SUPPLEMENTARY APPENDIX**

**PROGNOSTIC IMPLICATIONS OF IMPAIRED LONGITUDINAL LEFT VENTRICULAR SYSTOLIC FUNCTION ASSESSED BY TISSUE DOPPLER IMAGING PRIOR TO TRANSCATHETER AORTIC VALVE IMPLANTATION FOR SEVERE AORTIC STENOSIS**

Guglielmo Gallone MD^1^, Francesco Bruno MD^1^, Teresa Trenkwalder, MD^2^, Fabrizio D'Ascenzo MD PhD^1^, Fabian Islas MD^3^, Pier Pasquale Leone MD^4^, Philipp Nicol, MD^2^, Costanza Pellegrini, MD^2^, Enrico Incaminato^1^, Pilar Jimenez-Quevedo MD^3^, Hector Alfonso Alvarez-Covarrubias, MD^2^, Renato Bragato MD^4^, Alessandro Andreis MD^1^, Stefano Salizzoni MD^1^, Mauro Rinaldi MD, Prof^1^, Adnan Kastrati, MD, Prof^2^, Federico Conrotto MD^1^, Michael Joner MD, Prof^2^, Giulio Stefanini MD, Prof^4^, Luis Nombela-Franco MD^3^, Erion Xhepa MD, PhD^2^, Javier Escaned MD, Prof^3^, Gaetano M. De Ferrari MD, Prof^1^

1. Division of Cardiology, Department of Medical Sciences, , Città della Salute e della Scienza, University of Turin, Turin, Italy
2. Deutsches Herzzentrum München, Munich, Germany
3. Hospital Clínico San Carlos, IDISSC, and Universidad Complutense de Madrid, Madrid, Spain
4. Humanitas Clinical and Research Center IRCCS, Rozzano-Milan, Italy

**Corresponding author**:

Guglielmo Gallone, MD

Division of Cardiology, Città della Salute e della Scienza, Torino, Italy

Corso Bramante 88/90, 10126, Turin, Italy

Email: guglielmo.gallone@gmail.com Phone: +390116335443

1. **Study collaborators**

Filippo Angelini MD^1^, Pier Paolo Bocchino MD^1^, Francesco Piroli MD^1^, Giulia De Lio MD^1^, Francesca De Lio MD^1^, Andrea Angelini MD^1^, Luca Scudeler MD^1^, Chiara Bongiovanni^1^, Matteo D’Amica^1^, Ovidio De Filippo MD^1^

1. Cardiovascular and Thoracic Department, Città della Salute e della Scienza, Turin, and Department of Medical Sciences, University of Turin, Turin, Italy

**Supplementary Figure 1. Procedural outcomes according to average S’ status**

|  | **Average S'**  **<6.5 cm/s  (n=164)** | **Average S'**  **≥6.5 cm/s**  **(n=133)** | **P-value** |
| --- | --- | --- | --- |
|  | PERIPROCEDURAL OUTCOMES | | |
| **Valve Embolization (%)** | 0 (0) | 0(0) | 1.000 |
| **Second valve Implanted (%)** | 1 (1.1) | 0 (0) | 0.535 |
| **Annular Rupture (%)** | 1 (0.8) | 0 (0) | 0.551 |
| **Aortic dissection (%)** | 0 (0) | 0 (0) | 1.000 |
| **Pericardial Tamponade (%)** | 2 (1.2) | 1 (0.8) | 0.581 |
| **Coronary Occlusion (%)** | 1 (0.6) | 0 (0) | 0.554 |
| **Conversion to open heart surgery (%)** | 1 (0.6) | 1 (0.8) | 0.691 |
